# Effects of different protein sources on nutrient disappearance, rumen fermentation parameters and microbiota in dual-flow continuous culture system

**DOI:** 10.1186/s13568-022-01358-1

**Published:** 2022-02-10

**Authors:** Hui Mi, Ao Ren, Jinjia Zhu, Tao Ran, Weijun Shen, Chuanshe Zhou, Bin Zhang, Zhiliang Tan

**Affiliations:** 1 CAS Key Laboratory for Agro-Ecological Processes in Subtropical Region, National Engineering Laboratory for Pollution Control and Waste Utilization in Livestock and Poultry Production, Hunan Provincial Key Laboratory of Animal Nutritional Physiology and Metabolic Process, Institute of Subtropical Agriculture, Chinese Academy of Sciences, Changsha, 410125 Hunan People’s Republic of China; 2 College of Animal Science and Technology, Hunan Agricultural University, Changsha, 410128 Hunan People’s Republic of China; 3 College of Pastoral Agriculture Science and Technology, Lanzhou University, Lanzhou, 730000 Gansu People’s Republic of China; 4 University of Chinese Academy of Sciences, Beijing, 100049 People’s Republic of China

**Keywords:** Protein sources, Nutrient disappearance, Rumen fermentation parameters, Microbiota, Dual-flow continuous culture system

## Abstract

Scarce high-quality protein feed resources has limited the development of animal husbandry. In this study, we used a dual-flow continuous culture system to evaluate effects of difference dietary protein sources including soybean meal (SBM), cottonseed meal (CSM), and rapeseed meal (RSM), on nutrient disappearance, rumen fermentation, and microbiota of *XiongDong* black goats. Dietary proteins of either CSM, RSM or SBM had no effect on nutrient disappearance (*P* > 0.05). CSM or RSM significantly reduced (*P* < 0.01) the pH and enhanced (*P* < 0.01) the ammonia nitrogen (NH_3_-N) concentration in fermentation liquid compared to SBM. The short-chain fatty acids (SCFAs) contents were greater (*P* = 0.05) and acetate was lower (*P* < 0.01) in SBM than those in RSM and CSM, whereas propionate was greater (*P* < 0.01) in RSM than that in SBM, consequently reducing the acetate to propionate ratio (A/P) in RSM. Bacteroidetes, Firmicutes, and Proteobacteria were detected as the dominant phyla, and the relative abundances of Spirochaetae (*P* < 0.01) and Chlorobi (*P* < 0.05) declined in the CSM and RSM groups as compared to those in the SBM group. At the genus level, *Prevotella_1* was the dominant genus; as compared to SBM, its relative abundance was greater (*P* < 0.01) in CSM and RSM. The abundances of *Prevotellaceae_Ga6A1* and *Christensenellaceae_R7* were lower (*P* < 0.05) in CSM, whereas *Eubacterium_oxidoreducens_group*, and *Treponema_2* were lower (*P* < 0.01) in both CSM and RSM, and other genera were not different (*P* > 0.10). Although the bacterial community changed with different dietary protein sources, the disappearances of nutrients were not affected, suggesting that CSM and RSM could be used by rumen bacteria, as in case with SBM, and are suitable protein sources for ruminant diets.

## Introduction

Proteins derived from plants, animals, and non-protein nitrogen are commonly used as crude protein (CP) sources in ruminant diets. Soybean meal (SBM) has been the main protein source because of its relatively high concentration of CP and rumen-undegraded protein (RUP) and its well-balanced amino acid (AA) composition (Cherif et al. 2018). However, with the high and variable prices in recent years (Romero-Huelva et al. 2017), demand for alternatives to SBM to reduce feeding costs and enhance farm profits has increased (Cherif et al. 2018). Several agricultural byproducts such as rapeseed meal (RSM) and cottonseed meal (CSM), which are cost-effective and easily available, have recently been used as protein sources in ruminant diets.

Numerous researchers have investigated the influence of various protein sources on nutrient digestibility and rumen fermentation in ruminants (Cherif et al. 2018; Paula et al. 2020; Sanchez-Duarte et al. 2019). Proteins from various sources usually have different chemical characteristics, such as AA composition, contents of rumen degradable protein (RDP) and RUP, and the RDP/RUP. Differences in the chemical characteristics of diets are the main factors that affect digestibility, rumen fermentation characteristics and microbial exploitability of protein from different sources (Huang et al. 2019; Klevenhusen et al. 2017). Rumen fermentation and the microbial profile could be appropriately modified when goats were fed diets from different dietary protein sources, this could be due to the differences in the structure and degradability of different protein sources (Wang et al. 2009). The degradability of CP in the rumen is considerably related to the ruminal ammonia nitrogen (NH_3_-N) concentration, and McCarthy et al. (1989) reported a greater NH_3_-N concentration in SBM than that in corn gluten meal due to the higher ruminal degradability of SBM. Similarly, Cherif et al. (2018) reported that cows fed ground or rolled faba bean had higher ruminal NH_3_-N concentration than those fed SBM, which was because of higher solubility and degradability of CP in ground or rolled faba bean. However, protein sources have negligible effect on the digestibility of other nutrients in the rumen. McCarthy et al. (1989) found that digestion of organic matter (OM), starch, acid detergent fiber (ADF), and neutral detergent fiber (NDF) was not affected by dietary CP in the rumen. SBM and RSM could be partially replaced with feed grade urea or slow-release urea, without affecting milk performance or diet digestibility (Sinclair et al. 2012).

Both RSM and CSM are characterized to have a higher RUP content than that in SBM (NRC 2001). They are alternative sources of protein that can be used to replace SBM in ruminant diets. However, whether SBM replaced with CSM or RSM affects the production performance of animals remains uncertain, both negative (Imaizumi et al. 2016) and positive (Rutkowska et al. 2015; Shingfield et al. 2016) effects on ruminal fermentation and production performance have been observed when RSM and CSM were used in ruminant diets. We hypothesised that different alternative effects resulting from dietary protein sources might be associated with the transformation of the rumen microbiota. Numerous studies have highlighted that the type of diet can alter rumen microbiota (Cremonesi et al. 2018; Klevenhusen et al. 2017; Niu et al. 2017). Furthermore, studies have focused on the association between dietary protein and rumen microbiota. He et al. (2018) found the high CP diets were beneficial for the growth of *Butyrivibrio fibrisolvens* because of the greater level of ammonia in the rumen of Holstein bulls. Wang et al. (2019) suggested that gastrointestinal tract microbial communities influences the utilization efficiency of nitrogen in goats. Zhang et al. (2020b) reported that reducing dietary CP content by 3% diminished the relative richness of *Bacteroidetes,* which might be associated with the increasing efficiency of carbohydrate utilization in the rumen. Most previous studies have concentrated on how protein levels affect ruminal microbiota, and the influence of different plant protein sources on ruminal bacterial communities has relatively not been examined. Therefore, this study aimed to use a dual-flow continuous culture system to evaluate effects of different dietary protein sources including SBM, CSM or RSM on nutrient disappearance, rumen fermentation and microbiota..

## Materials and methods

This study was approved by the Animal Care Committee of the Institute of Subtropical Agriculture, Chinese Academy of Sciences, and the College of Animal Science and Technology, Hunan Agricultural University, Changsha, China.

### Continuous culture system

We used a dual-flow continuous culture fermentation system because continuous culture fermenters run continuously for longer periods than in the Daisy^II^, batch culture and the Ankon Gas Production System, furthermore, the continuous culture can mimic in vivo ruminal conditions and allows for natural stratification of feed particles, anaerobiosis, controlled temperature, and salivary buffering, similar to what occurs in the rumen (Alende et al. 2018). A dual-flow continuous culture system (Adebayo Arowolo et al. 2021) was used in this study. This system contains six fermentation units, each unit consisting of a fermentation apparatus, outflow device, liquid supply component, data detection module and controlling computer. The fermenter, with a working volume of 1000 ± 20 mL, is the center of each fermentation unit, and various sensors are installed on the fermenters to monitor the pressure, pH value and temperature in real time. The overflow device is connected to the overflow pipe of the fermenter. A solid discharger is installed at the bottom of the fermenter, and the gas flow meter, and CO_2_ and CH_4_ detectors are installed on the exhaust path to measure the exhaust volume and the CO_2_ and CH_4_ concentration in the fermenters used in this study (Shen et al. 2012).

### Experimental design and treatments

The experiment was a replicated 3 × 3 Latin square design that evolved a dual-flow continuous culture fermentation system with six fermenters. The experimental diets were formulated and prepared as a total mixed ration (TMR), containing a forage: concentrate ratio of 50:50 (Table 1). Three different protein sources were used with the same CP content (12.26%): SBM, RSM, and CSM. This study contained three periods and each period lasted 8 days, which included 5 days for adaptation and 3 days for sampling.

**Table 1:** Composition and nutrient levels of basal diets (DM basis)

Item	Treatments		
	SBM	RSM	CSM
Feed ingredients, % of DM			
Corn	31.50	30.00	30.00
Soybean meal	16.00	/	/
Rapeseed meal	/	19.04	/
Cottonseed meal	/	/	16.50
Maize straw	50.00	50.0	50.00
CaCO_3_	0.24	0.00	0.00
Ca_3_(PO4)_2_	0.68	0.79	1.17
NaCl	0.60	0.60	0.60
Premix^a^	1.00	1.00	1.00
Chemical composition (%)			
ME (Mcal/kg)	2.19	2.19	2.19
CP	12.26	12.26	12.26
RUP	7.10	7.96	7.52
RDP	6.98	3.96	3.89
SC	33.83	37.05	36.55
NDF	36.24	37.11	38.09
ADF	25.89	26.64	26.74
Ca	0.51	0.50	0.49
P	0.32	0.43	0.44

ME: Metabolic energy; CP: Crude protein; RUP, Rumen undegradable protein; SC: Structural carbohydrates; NDF: Neutral detergent fiber; ADF: Acid detergent fiber; Ca: Calcium; P: Phosphorus; RDP: Rumen degradable protein; SBM: Soybean meal; RSM: Rapeseed meal; CSM: Cottonseed meal

^a^The premix provided (per kg of the diet): MgSO_4_·H_2_O 119 mg, FeSO_4_·H_2_O1.53 mg, CuSO_4_·5H_2_O 0.8 mg, MnSO_4_·H_2_O 3 mg, ZnSO_4_·H_2_O 5 mg, Na_2_SeO_3_ 10 mg, KI 40 mg, CoCl_2_·6H_2_O 30 mg, VA 95 000 IU, VD17 500 IU and VE 18 000 IU

Three *Xiongdong* black goats (with an average body weight of 25.0 ± 2.5 kg) with permanent ruminal fistulas were used as rumen fluid donors. The goats were offered a TMR (500 g/kg maize straw, 315 g/kg corn, 160 g/kg soybean meal, 2.4 g/kg calcium carbonate, 6.8 g/kg calcium phosphate, 6.0 g/kg sodium chloride, and 10 g/kg premix with vitamins and microelements) twice daily in equal amounts (300 g per meal) at 08:00 and 18:00 h. Donor animals had free access to water and were adapted to diets 14 days before rumen fluid donation. Rumen contents were collected via the rumen cannula before morning feeding, and immediately transported to the laboratory in an insulated bottle pre-warmed with warm water (39.5 ± 0.5 °C). Then the rumen contents were squeezed through four layers of cheesecloth and mixed well. The pH was recorded, and samples were kept in a water bath with continuous release of CO_2_ prior to introducing into fermentation vessels.

Before starting the formal experiment, the inner surfaces of the fermentation vessels, pH meter, thermometer and mixing propeller were disinfected with 75% ethanol and then assembled ensure a gastight condition. To initiate fermentation, 500 mL of artificial saliva (which contained: 9.8 g NaHCO_3_, 9.3 g Na_2_PO_4_·12H_2_O, 0.47 g NaCl, 0.57 g KCl, 0.12 g Mg·7H_2_O and 0.04 g CaCl_2_ per litter) (McDougall 1949) and 500 mL rumen inoculum were added to each fermenter through the feeder nose, and 20 g of the corresponding experimental diet was fed to each fermenter at 08:00 h on the first day of each period. The whole feeding process was performed with continuous N_2_ bubbling into fermenters to exhaust air (Brandao et al. 2018). After inoculation to fermenters, artificial saliva (McDougall 1949) was infused to maintain a liquid dilution rate of 6%/h. The effluent flow out of the fermenters was ~ 1440 mL per day. The temperature was kept constant at 39.5 ± 0.5 ℃ using warm water, and the rotation speed of the mixing propeller was set at 25 rpm to simulate rumen peristalsis. Thereafter, 40 g of the diets were fed in two equal meals (2 × 20 g) at 08:00 h and 20:00 h on each day of the experimental period (Zhang et al. 2020a).

### Sample collection procedures

At 08:00 h on days 6, 7 and 8, samples were collected from total effluent before feeding and all samples were composited for three days (Fowler et al. 2015) for short-chain fatty acid (SCFAs) and NH_3_-N analysis. Briefly, 1.5-mL of effluent was acidified using 0.15 mL 25% (w/v) metaphosphoric acid for analysis of SCFAs, and 1.5 mL of effluent was acidified with 0.15 mL H_2_SO_4_ (1% vol/vol) for NH_3_-N analysis. All samples were stored at − 20 °C. Another 1.5-mL of effluent sample was collected from each fermenter on each sampling day and stored at − 80 °C, samples of three days were composited for DNA extraction(Firkins et al. 2015).The remaining effluent was handled to collect residuals for determining the in vitro nutrient disappearance using the method described by Li et al.(2016). The pH values were recorded using a pH meter inside the fermenter at 08:00 h before feeding on each of the sampling days, and 5 mL of fermentation fluid was collected from each fermenter into a container that containing 10 mL of methyl green solution (containing 1 mL methanol, 0.08 g NaCl and 0.006 g methyl green) for protozoa counting (Shen et al. 2012).

### Chemical analysis

The total nitrogen (TN) content was analyzed according to the methods of AOAC (2002) and the CP content was calculated as TN × 6.25. NDF and ADF were determined as reported by Van Soest et al. (1991) and AOAC (2002), respectively. The dry matter (DM) contents of residual feed, DM disappearance (DMD), NDF disappearance (NDFD) and ADF disappearance (ADFD) were determined following the procedures described by Zhang et al. (2018). The SCFAs concentration was measured using gas chromatography according to the procedure described by Harvatine et al. (2002), and the NH_3_-N concentration was determined using phenol-hypochlorite reaction method of Weatherburn (1967). For protozoa population counting, 1 mL mixed liquor was transferred to Sedgewick-Rafter counting plate (Arthur Thomas no. 9851-C20) to count the protozoa population using a microscope with 100× magnification, the counting chamber is consisted of 1000 grids, and the total number of protozoa in 25 randomly selected grids was recorded as N using a five-point sampling method. The population of protozoa in fermenters were calculated by the following equation:

$${\text{Protozoa population }} = {\text{ N}} \times ({1}000/{25}) \times {3}$$

### DNA extraction and sequencing

Total genomic DNA was extracted using DNA Extraction Kit (Qiagen, Germany) following the manufacturer’s instructions. The quality and quantity of DNA were verified using NanoDropND1000 (NanoDrop Technologies, Inc, Wilmington, DE, USA) and agarose gel. Extracted DNA was diluted to a concentration of 1 ng/μL and used as templates for PCR amplification of bacterial 16S rRNA genes. The V3-V4 variable regions of 16S rRNA genes were amplified with universal primers 343F (5′-TACGGRAGGCAGCAG-3′) and 798R (5′-AGGGTATCTAATCCT-3′), with different barcodes for each sample. The amplicon was visualized using gel electrophoresis, purified with AM Pure XP beads (Agencourt), and quantified using the Qubitds DNA assay kit. Bar-coded amplicons were mixed at equal molar ratios and subjected to Illumina paired-end library preparation, cluster generation, and 300-bp paired-end sequencing on an Illumina MiSeq PE300 instrument.

### Bioinformatic analysis

Paired-end reads were preprocessed using Trimmomatic software (Bolger et al. 2014) to detect and cut off ambiguous bases (N), and low-quality sequences. After trimming, paired-end reads were assembled using FLASH software (Reyon et al. 2012). The assembly parameters were: 10 bp of minimal overlapping, 200 bp of maximum overlapping and 20% of maximum mismatch rate. Reads with chimera were detected and removed using the QIIME software (version 1.8.0) (Caporaso et al. 2010). Clean reads were subjected to primer sequence removal and clustered to generate operational taxonomic units (OTUs) using UPARSE with 97% similarity cutoff (Edgar 2013). The representative reads of each OTU were selected using the QIIME package. All representative reads were annotated against Silva database (version 123) using the RDP classifier (Wang et al. 2007). Taxonomic identification and comparisons were performed at phylum and genus levels. Alpha diversity values were obtained using various diversity indices as described by Jiao et al. (2015). Principal coordinate analysis (PCoA) of the microbial communities was performed using the weighted UniFrac distance.

### Statistical analysis

A 3 × 3 Latin square design with repetition was used in this study. All data were analyzed using SAS 9.2 (SAS 2009). Data on nutrient disappearance, fermentation parameters, protozoa counts, alpha diversity indices, relative abundances of bacteria at the phylum and genus levels were checked for normality (Kolmogorov-Smirov test) and variance homogeneity (F-test) before further statistical analysis. For variables that did not met the assumptions required for ANOVA (alpha diversity indices, relative abundances of bacteria), non-parametric test models were applied (Kruskal–Wallis procedure of SAS). For variables that meet the assumptions required for ANOVA, the MIXED procedure was used to analyze the pH and protozoa counts, the model included the fixed effect of treatments, period, fermenter, sampling time and the interaction between treatment and sampling time. Sampling time was considered as the repeated measure, and the fermenter and experimental period were random effects. The nutrient disappearance, SCFAs and NH_3_-N levels were analyzed using one-way ANOVA. Statistical significance was accepted at *P* < 0.05 and 0.05 ≤ *P* ≤ 0.1 was considered as a trend.

## Results

### In vitro* nutrient disappearance*

The in vitro nutrient disappearance is shown in Table 2. The DMD, ranging from 78.59% to 79.41%, did not differ among treatments (*P* > 0.10); CPD was also not affected (*P* > 0.10) by treatments, but the CPD in the SBM group was 10.68% numerically higher than that in the CSM group. Both NDFD and ADFD were not affected with different dietary protein sources in terms of fiber content (*P* > 0.10).

**Table 2:** Nutrients digestibility affected by different protein source of diets

Item	Treatments			SEM	*P*-Value
	SBM	RSM	CSM		
DMD (%)	78.78	78.59	79.41	1.24	0.98
CPD (%)	77.89	70.37	75.71	2.01	0.25
NDFD (%)	63.52	63.03	61.98	2.40	0.98
ADFD (%)	73.47	71.85	74.31	1.79	0.93

DMD: Dry matter disappearance; CPD: Crude protein disappearance; NDFD: Neutral detergent fiber disappearance; ADFD: Acid detergent fiber disappearance; SBM: Soybean meal; RSM: Rapeseed meal; CSM: Cottonseed meal

### Fermentation parameters and protozoa counting

As presented in Table 3, the SBM treatment had significantly higher pH value (*P* < 0.01) and total SCFAs contents (*P* = 0.05) but lower (*P* < 0.01) NH_3_-N concentrations than those in RSM and CSM treatments, with no differences observed between RSM and CSM (*P* > 0.05). The RSM treatment had a lower molar proportion of acetate (*P* < 0.01) and acetate to propionate ratio (A/P) (*P* < 0.01) and a greater proportion of propionate (*P* < 0.01) and butyrate (*P* < 0.01) than those obtained from SBM and CSM treatment. The content of branch-chained SCFAs in the CSM group was lower (*P* < 0.01) than that of SBM and RSM groups, whereas the valerate content did not differ among treatments (*P* > 0.10). The SBM treatment tended to have a greater (*P* = 0.09) protozoa enumeration than that from the RSM and CSM treatments (Table 3).

**Table 3:** Rumen fermentation parameters affected by different protein source of diets

Item	Treatments			SEM	*P*-Value
	SBM	RSM	CSM		
pH	6.84^a^	6.68^b^	6.62^b^	0.03	< 0.01
NH_3_-N(mg/dL)	3.10^b^	4.63^a^	5.46^a^	0.38	< 0.01
Protozoa, × 10^3^cells/mL	19.04	15.19	14.01	0.98	0.09
Acetate (%)	68.14^a^	65.19^b^	68.06^a^	0.80	< 0.01
Propionate (%)	15.85^b^	18.30^a^	16.50^b^	0.62	< 0.01
Butyrate (%)	10.63^b^	13.04^a^	11.71^b^	1.30	< 0.01
Valerate (%)	2.18	1.80	1.84	0.12	0.33
Branch-chained SCFAs	2.38^a^	2.25^a^	1.88^b^	0.06	< 0.01
Total SCFAs (mM)	119.73^a^	102.13^b^	108.61^b^	6.03	0.05
Acetate/Propionate	4.34^a^	3.58^b^	4.16^a^	0.11	< 0.01

SBM: Soybean meal; RSM: Rapeseed meal; CSM: Cottonseed meal

^a, b^Mean values within a row with unlike superscript letters were significantly different (*P* < 0.05)

### Alpha and beta diversity

**Fig. 1:**
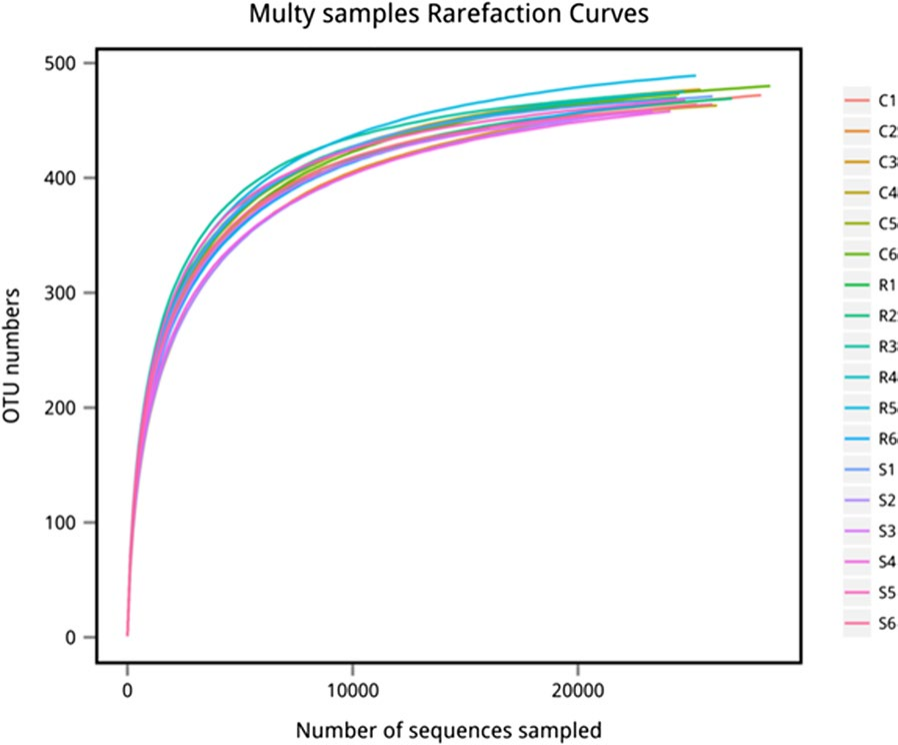
The rarefaction curve of species. (C1-C6 indicated samples in CSM, R1-R6 indicated samples in RSM, S1-S6 indicated samples in SBM.)

**Fig. 2:**
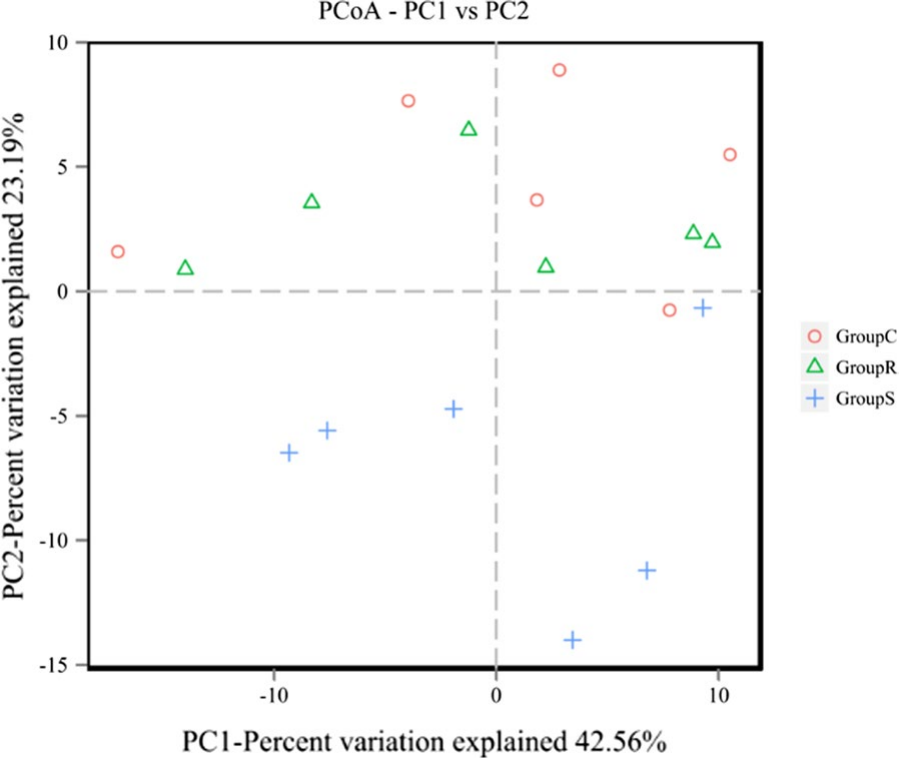
Principal coordinate analysis (PCoA) of the ruminal bacterial OTUs in different protein source diets. (Group C showed samples in CSM, Group R showed samples in RSM, Group S showed samples in SBM.)

The rarefaction curves (Fig. 1) indicated that all of our sampling efforts provided sufficient OTU coverage to accurately describe the bacterial composition of each group. The results of alpha diversity indices (Table 4) showed that the OTU number of RSM was significantly greater than that of SBM (*P* = 0.03); chao and ACE indices tended to be greater (0.05 < *P* < 0.10) in RSM. However, the Shannon, Simpson and coverage indices (*P* > 0.10) among three treatments were not significantly different. PCoA revealed that the samples of RSM and CSM treatment clustered and were separated from those of the SBM treatment, indicating that the bacterial diversity in the SBM group was different from that in RSM and CSM groups (Fig. 2).

**Table 4:** Species richness, diversity and evenness indices of rumen bacteria affected by different protein source of diets

	Treatments			SEM	*P*-value
	SBM	RSM	CSM		
OUTs number	527.83^b^	545.67^a^	536.00^ab^	2.94	0.03
Chao	566.53	587.82	572.80	5.91	0.07
Shannon	6.83	6.84	6.77	0.10	0.86
Simpson	0.98	0.97	0.97	< 0.01	0.75
Coverage	1.00	1.00	1.00	< 0.01	0.80
ACE observed species	523.67	539.00	528.67	4.57	0.08
PD_whole_tree	22.14	22.69	22.06	0.23	0.14

SBM: Soybean meal; RSM: Rapeseed meal; CSM: Cottonseed meal

^ab^Mean values within a row with unlike superscript letters were significantly different (*P* < 0.05)

### Bacterial taxonomy at the phylum and genus levels

In all, 13 phyla were identified within the ruminal bacteria (Table 5), with the most dominant phyla being Bacteroidetes (59.37%), Firmicutes (23.06%), Spirochaetae (9.85%), and Proteobacteria (5.71%). The relative abundances of Spirochaetae (*P* < 0.01) *and* Chlorobi (*P* = 0.02) were lower in the RSM and CSM groups than in the SBM group. Meanwhile, the relative abundance of Actinobacteria tended to be greater (*P* = 0.06) in the RSM group than in the SBM group. The relative abundances of the other phyla did not differ among the three treatments (*P* > 0.10).

**Table 5:** Effects of different dietary protein source on the relative abundance (%) of the ruminal bacterial at phylum level

Taxon	Treatments			SEM	*P*-value
	SBM	RSM	CSM		
*Bacteroidetes*	57.53	58.60	62.00	1.58	0.44
*Firmicutes*	24.48	23.04	21.67	0.88	0.46
*Spirochaetae*	8.71^a^	4.14^b^	4.27^b^	0.64	0.01
*Proteobacteria*	7.39	11.84	10.31	1.49	0.46
*Fibrobacteres*	1.01	1.50	0.98	0.12	0.14
*Tenericutes*	0.52	0.48	0.39	0.04	0.28
*Lentisphaerae*	0.12	0.12	0.11	0.01	0.81
*Elusimicrobia*	0.12	0.14	0.19	0.03	0.91
*Chlorobi*	0.04^a^	0.01^b^	0.01^b^	0.01	0.02
*Actinobacteria*	0.01	0.03	0.01	0.00	0.06
*Cyanobacteria*	0.00	0.01	0.02	0.00	0.55
*Saccharibacteria*	0.00	0.00	0.00	0.00	0.14
*Others*	0.06	0.09	0.05	0.01	0.22

SBM: Soybean meal; RSM: Rapeseed meal; CSM: Cottonseed meal

^ab^Mean values within a row with unlike superscript letters were significantly different (P < 0.05)

Among all the genera detected, only five genera significantly changed with different dietary protein sources (Table 6). Within the *Bacteroidetes* phylum, the relative abundances of *Prevotella_1* in the CSM and RSM groups was increased by 42.23% and 32.89%, respectively, compared with those in the SBM group (*P* < 0.01). The relative abundance of *Prevotellaceae_Ga6A1_group*was lower (*P* = 0.03) in the CSM group than in the SBM and CSM groups. The relative abundance of *Prevotellaceae_UCG_003*showed a significant difference (*P* < 0.01) among the three treatments, in the order of CSM > RSM > SBM. Within the *Firmicutes* phylum, the relative abundance of *Eubacterium_oxidoreducens* in the CSM and RSM groups was lower (*P* < 0.01) than that in the SBM group. Meanwhile, the relative abundance of *Christensenellaceae_R_7* in the CSM group was lower (*P* = 0.03) than that in the SBM and RSM treatments. In the phylum *Spirochaetae*, *Treponema_2* exhibited a relatively higher abundance in the SBM group than in the other two groups (*P* < 0.01).

**Table 6:** Relative abundance (%) of the most 30 abundant bacterial at genus level affected by different protein source of diets

Taxon		Treatments			SEM	*P*-value
Phylum	Genus	SBM	RSM	CSM		
*Bacteroidetes*	*Prevotellaceae_UCG_003*	0.93^c^	1.39^b^	2.22^a^	0.15	< 0.01
	*Prevotella_1*	18.72^b^	24.88^a^	26.62^a^	1.16	0.01
	*Prevotellaceae_Ga6A1_group*	1.20^a^	1.13^a^	0.47^b^	0.13	0.03
	*Prevotellaceae_UCG_001*	0.96	0.82	1.25	0.08	0.14
	*U29_B03*	0.25	0.47	0.27	0.05	0.17
	*Haemonchus_placei*	0.82	0.97	1.19	0.14	0.50
	*Rikenellaceae_RC9_gut_group*	7.40	7.61	7.93	0.50	0.83
	*Bacteroides*	1.30	1.59	1.24	0.21	0.85
*Firmicutes*	*[Eubacterium]_oxidoreducens_group*	0.85^a^	0.18^b^	0.31^b^	0.10	0.01
	*Christensenellaceae_R_7_group*	0.75^a^	0.85^a^	0.41^b^	0.07	0.03
	*Ruminococcaceae_UCG_010*	0.37	0.58	0.46	0.04	0.09
	*Ruminococcus_1*	1.77	2.28	2.07	0.11	0.13
	*Family_XIII_AD3011_group*	1.32	1.11	0.65	0.14	0.16
	*Ruminiclostridium_5*	0.89	0.66	0.69	0.05	0.19
	*Butyrivibrio_2*	0.43	0.39	0.35	0.02	0.24
	*[Eubacterium]_coprostanoligenes_group*	0.82	0.65	0.67	0.05	0.31
	*Lachnospiraceae_NK4A136_group*	0.88	0.94	0.92	0.06	0.33
	*Papillibacter*	0.31	0.37	0.30	0.04	0.50
	*Ruminococcaceae_UCG_014*	3.24	3.64	3.98	0.26	0.53
	*Anaerosporobacter*	0.54	0.54	0.83	0.20	0.63
	*Ruminococcaceae_UCG_005*	0.79	0.68	0.77	0.06	0.83
	*Ruminococcaceae_UCG_002*	0.78	0.68	0.77	0.11	0.75
	*Saccharofermentans*	0.51	0.56	0.61	0.04	0.88
	*Ruminococcaceae_NK4A214_group*	0.72	0.74	0.73	0.05	0.96
*Proteobacteria*	*Thalassospira*	0.90	0.83	0.60	0.10	0.28
	*Succinivibrionaceae_UCG_002*	0.34	0.20	0.57	0.15	0.35
	*Succinimonas*	4.98	8.61	7.73	1.42	0.57
	*Ruminobacter*	0.27	0.57	0.22	0.12	0.70
*Spirochaetae*	*Treponema_2*	8.14^a^	3.68^b^	3.83^b^	0.62	0.01
*Fibrobacteres*	*Fibrobacter*	0.99	1.48	0.95	0.12	0.14

SBM: Soybean meal; RSM: Rapeseed meal; CSM: Cottonseed meal

^ab^Mean values within a row with unlike superscript letters were significantly different(*P* < 0.05)

## Discussion

### *Effects of dietary protein sources on *in vitro* nutrient disappearance*

In the current study, DMD, CPD, NDFD, and ADFD were not different among SBM, RSM and CSM treatments when an in vitro dual-flow continuous culture fermentation system was used. Our results agreed with those reported in the in vivo study by McCarthy (1989) that digestion of DM, ADF, and NDF in the rumen was not affected by dietary CP sources. RSM and CSM were reported to have lower RUP content than SBM (NRC 2001), Paula et al. (2017) found that SBM or canola meal with RUP ranging from 38 to 50% of CP did not affect nutrient digestion. When fed at the maintenance level, Zagorakis et al (2018) reported that sheep received diets used SBM, RSM and pea seeds as protein sources had similar digestibility of DM, CP, NDF, and ADF. In a more recent study, Tian et al. (2019) reported that the type of dietary protein source (SBM, CSM, or RSM) had no effect on the effective degradation rate of DM and CP in dairy cows. The main difference in dietary nutrient composition in this study was the RDP content. Sun et al. (2019) reported that reducing the level of RDP in iso-nitrogenous diets had no effect on the apparent nutrient total-tract digestibility. All these studies suggested that RSM and CSM contained similar protein values and availability as SBM when included in ruminant diets. Moreover, this also suggested that the dual-flow continuous culture fermentation system is a feasible technology to mimic in vivo conditions.

### Effects of dietary protein sources on fermentation characteristics and protozoa population

Rumen fermentation can be modified properly by different sources of dietary protein (Wang et al. 2009). Higher RDP diets was expected to result in higher NH_3_-N concentrations (Agle et al. 2010); however, other studies have shown contrary results (Colmenero and Broderick 2006; Zhou et al. 2019), suggesting that RDP content was not always the main causes of NH_3_-N concentration, and that energy level and microbial protein synthesis efficiency also affect the NH_3_-N utilization (Russell et al. 1983). Besides, SBM treatment led to a greater total SCFAs concentration than CSM and RSM treatments, which were in accordance with Xu (2004). This is because RDP can either be used for microbial protein synthesis or be converted to SCFAs or NH_3_-N, which was unpredictable in the rumen (Firkins et al. 2007). However, if less RDP is used for SCFAs production and microbial protein synthesis due to energy limitations, more RDP will be converted to NH_3_-N, resulting in NH_3_-N accumulation (Russell et al. 1983). Taken together, we speculated that SBM was partially fermented to produce SCFAs instead of NH_3_-N, thus leading to greater SCFAs production by SBM treatment than CSM or RSM treatments. The differences in acetate and propionate percentages and acetate/propionate indicated that SBM and CSM diets were beneficial for fatty acids synthesis and RSM diet was benefit for protein synthesis (Sutton and Morant 1989). Branched-chain SCFAs (isobutyrate and isovalerate) were reported to derived from dietary branched-chain amino acids (Hobson and Stewart 1997), which accounted for 17.08%, 16.11% and 13.22% of CP in soybean, rapeseed, and cottonseed, respectively (NRC 2001). Thus, in the current study, the lower molar proportion of branched-chain SCFAs in CSM treatment was likely due to the lower content of branched-chain amino acids in CSM, Which had the same tendancy as protozoa counts. Some protozoal species such as entodiniomorphid protozoa engulf starch and sequester it from bacteria preventing its rapid fermentation by lactic acid producing bacteria (Bonhomme 1990), this indicates that protozoa may be involed in pH regulation. In adult ruminants, ciliate protozoa account for the dominance of protozoa in the rumen (Fonty et al. 1988). Additionally, ciliates can phagocytose bacteria and fungi, which can use NH_3_-N to synthesize microbial proteins and promote their propagation (Chalupa 1976). This might be the reason why the number of protozoa in the SBM group tended to be higher than those in the RSM and CSM groups.

### Effects of dietary protein sources on rumen microbiota

In all treatments, *Firmicutes, Bacteroidetes* and *Proteobacteria* were the dominant phyla, which was in accordance with the reports in dairy cows, goats, and beef cattle, regardless of the diet fed (Bickhart and Weimer 2018; Cremonesi et al. 2018; Zhou et al. 2019). This is because a core rumen microbial community is shared by ruminants, although the rumen microbial community composition varies with diet and host (Henderson et al. 2015). PCoA clustered the samples of different diet groups, suggesting that protein sources could affect the microbiota in the rumen. Notably, a greater relative abundance of *Spirochaetae* at the phylum level was observed in the SBM than in the RSM and CSM. *Spirochetes* plays an important role in degrading plant polymers materials such as xylan, pectin and arabinogalactan (Paster and Canale-Parola 1982). Pectin contents was higher in SBM (1.6%, 0.5% and 0.4% in SBM, RSM and CSM, respectively) (Zhu 2017). Thus, the difference of *Spirochetes* (*Treponema_2* in particular) might associate with pectin content.

At the genus level, *Prevotella_1* was the predominant genus, and its relative abundance was lower in SBM treatment when compared to RSM and CSM. The *Prevotella* genus has a wide metabolic niche due to genetic relatedness or high genetic variability that enables it to occupy different ecological niches within the rumen (Liu et al. 2019); thus; strains from the *Prevotella* genus are sensitive to dietary protein source. Another notable phenomenon lies in the fact that the *Eubacterium_oxidoreducens_group,* which belongs to *Firmicutes* phylum, was reduced in both the CSM and RSM groups.In the rumen, *Eubacterium* is associated with hemicellulolytic activity and usually produces organic acids (Taguchi et al. 2004). Because the NDF and ADF contents were similar in three diets, the surge in its abundance in the SBM diet might suggest that its potential role in protein degradation.

In conclusion, dietary protein sources could maintain stable ruminal fermentation, and the microbiota structure and relative abundance. Therefore, we believe that CSM and RSM are suitable protein sources in ruminant diets.

## Data Availability

The rumen fluid 16S rRNA-Seq data from this study have been submitted to the Sequence Read Archive (SRA) database (http://www.ncbi.nlm.nh.gov/sra) and the data are accessible through SRA Series accession number PRJNA758838 (http://www.ncbi.nlm.nih.gov/bioproject/758838).

## Abbreviations

CP: Crude protein
SBM: Soybean meal
RUP: Rumen-undegraded protein
AA: Amino acid
RSM: Rapeseed meal
CSM: Cottonseed meal
RDP: Rumen degradable protein
NH_3_-N: Ammonia nitrogen
OM: Organic matter
ADF: Acid detergent fiber
NDF: Neutral detergent fiber
TMR: Total mixed ration
SCFAs: Short-chain fatty acid
DM: Dry matter
DMD: DM disappearance
TN: Total nitrogen
NDFD: NDF disappearance
ADFD: ADF disappearance
OTUs: Operational taxonomic units
PcoA: Principal coordinate analysis
